# Supplementary material for: HIVprotI: an integrated web based platform for prediction and design of HIV proteins inhibitors
Source: J Cheminform. 2018 Mar 9;10:12. doi: 10.1186/s13321-018-0266-y (PMC5845081; doi:10.1186/s13321-018-0266-y)
Supplement: Supplementary file 2 — Additional file 2. Source code of HIVProtI web server. [file 13321_2018_266_MOESM2_ESM.zip › HIVprotI_Source-code/hivprotI/weka-3-6-6/doc/weka/gui/ConverterFileChooser.html]

ConverterFileChooser


---


|  |  |  |
| --- | --- | --- |
| **Overview** \| **Package** \| **Class** \| **Tree** \| **Deprecated** \| **Index** \| **Help** \| **Weka's home** | |  |
| **PREV CLASS**   **NEXT CLASS** | **FRAMES**    **NO FRAMES**     **All Classes** |  |
| SUMMARY: NESTED \| FIELD \| CONSTR \| METHOD | DETAIL: FIELD \| CONSTR \| METHOD |  |


---


## weka.gui Class ConverterFileChooser

```
java.lang.Object
  java.awt.Component
      java.awt.Container
          javax.swing.JComponent
              javax.swing.JFileChooser
                  weka.gui.ConverterFileChooser
```

**All Implemented Interfaces:**: java.awt.image.ImageObserver, java.awt.MenuContainer, java.io.Serializable, javax.accessibility.Accessible

---

``` public class ConverterFileChooser extends javax.swing.JFileChooser ```

A specialized JFileChooser that lists all available file Loaders and Savers.
To list only savers that can handle the data that is about to be saved, one
can set a Capabilities filter.

**Version:**
:   $Revision: 7464 $

**Author:**
:   fracpete (fracpete at waikato dot ac dot nz)

**See Also:**: `setCapabilitiesFilter(Capabilities)`, Serialized Form

---

**Nested Class Summary**

**Nested classes/interfaces inherited from class javax.swing.JComponent**

`javax.swing.JComponent.AccessibleJComponent`

**Nested classes/interfaces inherited from class java.awt.Component**

`java.awt.Component.BaselineResizeBehavior`


| **Field Summary** | |
| --- | --- |
| `static int` | `LOADER_DIALOG`             the loader dialog |
| `static int` | `SAVER_DIALOG`             the saver dialog |
| `static int` | `UNHANDLED_DIALOG`             unhandled type of dialog |

**Fields inherited from class javax.swing.JFileChooser**

`ACCEPT_ALL_FILE_FILTER_USED_CHANGED_PROPERTY, ACCESSORY_CHANGED_PROPERTY, APPROVE_BUTTON_MNEMONIC_CHANGED_PROPERTY, APPROVE_BUTTON_TEXT_CHANGED_PROPERTY, APPROVE_BUTTON_TOOL_TIP_TEXT_CHANGED_PROPERTY, APPROVE_OPTION, APPROVE_SELECTION, CANCEL_OPTION, CANCEL_SELECTION, CHOOSABLE_FILE_FILTER_CHANGED_PROPERTY, CONTROL_BUTTONS_ARE_SHOWN_CHANGED_PROPERTY, CUSTOM_DIALOG, DIALOG_TITLE_CHANGED_PROPERTY, DIALOG_TYPE_CHANGED_PROPERTY, DIRECTORIES_ONLY, DIRECTORY_CHANGED_PROPERTY, ERROR_OPTION, FILE_FILTER_CHANGED_PROPERTY, FILE_HIDING_CHANGED_PROPERTY, FILE_SELECTION_MODE_CHANGED_PROPERTY, FILE_SYSTEM_VIEW_CHANGED_PROPERTY, FILE_VIEW_CHANGED_PROPERTY, FILES_AND_DIRECTORIES, FILES_ONLY, MULTI_SELECTION_ENABLED_CHANGED_PROPERTY, OPEN_DIALOG, SAVE_DIALOG, SELECTED_FILE_CHANGED_PROPERTY, SELECTED_FILES_CHANGED_PROPERTY`

**Fields inherited from class javax.swing.JComponent**

`TOOL_TIP_TEXT_KEY, UNDEFINED_CONDITION, WHEN_ANCESTOR_OF_FOCUSED_COMPONENT, WHEN_FOCUSED, WHEN_IN_FOCUSED_WINDOW`

**Fields inherited from class java.awt.Component**

`BOTTOM_ALIGNMENT, CENTER_ALIGNMENT, LEFT_ALIGNMENT, RIGHT_ALIGNMENT, TOP_ALIGNMENT`

**Fields inherited from interface java.awt.image.ImageObserver**

`ABORT, ALLBITS, ERROR, FRAMEBITS, HEIGHT, PROPERTIES, SOMEBITS, WIDTH`


**Constructor Summary**

`ConverterFileChooser()`
  
          onstructs a FileChooser pointing to the user's default directory.

`ConverterFileChooser(java.io.File currentDirectory)`
  
          Constructs a FileChooser using the given File as the path.

`ConverterFileChooser(java.lang.String currentDirectory)`
  
          Constructs a FileChooser using the given path.


| **Method Summary** | |
| --- | --- |
| `Capabilities` | `getCapabilitiesFilter()`             returns the capabilities filter for the savers, can be null if all are listed. |
| `boolean` | `getCoreConvertersOnly()`             Returns whether only the hardcoded core converters are displayed. |
| `boolean` | `getFileMustExist()`             Returns whether the selected file must exist (only open dialog). |
| `AbstractFileLoader` | `getLoader()`             returns the loader that was chosen by the user, can be null in case the user aborted the dialog or the save dialog was shown |
| `boolean` | `getOverwriteWarning()`             Returns whether a popup appears with a warning that the file already exists (only save dialog). |
| `AbstractFileSaver` | `getSaver()`             returns the saver that was chosen by the user, can be null in case the user aborted the dialog or the open dialog was shown |
| `static void` | `main(java.lang.String[] args)`             For testing the file chooser |
| `void` | `setCapabilitiesFilter(Capabilities value)`             sets the capabilities that the savers must have. |
| `void` | `setCoreConvertersOnly(boolean value)`             Whether to display only the hardocded core converters. |
| `void` | `setFileMustExist(boolean value)`             Whether the selected file must exst (only open dialog). |
| `void` | `setOverwriteWarning(boolean value)`             Whether a warning is popped up if the file that is to be saved already exists (only save dialog). |
| `int` | `showDialog(java.awt.Component parent, java.lang.String approveButtonText)`             Pops a custom file chooser dialog with a custom approve button. |
| `int` | `showOpenDialog(java.awt.Component parent)`             Pops up an "Open File" file chooser dialog. |
| `int` | `showSaveDialog(java.awt.Component parent)`             Pops up an "Save File" file chooser dialog. |

**Methods inherited from class javax.swing.JFileChooser**

`accept, addActionListener, addChoosableFileFilter, approveSelection, cancelSelection, changeToParentDirectory, ensureFileIsVisible, getAcceptAllFileFilter, getAccessibleContext, getAccessory, getActionListeners, getApproveButtonMnemonic, getApproveButtonText, getApproveButtonToolTipText, getChoosableFileFilters, getControlButtonsAreShown, getCurrentDirectory, getDescription, getDialogTitle, getDialogType, getDragEnabled, getFileFilter, getFileSelectionMode, getFileSystemView, getFileView, getIcon, getName, getSelectedFile, getSelectedFiles, getTypeDescription, getUI, getUIClassID, isAcceptAllFileFilterUsed, isDirectorySelectionEnabled, isFileHidingEnabled, isFileSelectionEnabled, isMultiSelectionEnabled, isTraversable, removeActionListener, removeChoosableFileFilter, rescanCurrentDirectory, resetChoosableFileFilters, setAcceptAllFileFilterUsed, setAccessory, setApproveButtonMnemonic, setApproveButtonMnemonic, setApproveButtonText, setApproveButtonToolTipText, setControlButtonsAreShown, setCurrentDirectory, setDialogTitle, setDialogType, setDragEnabled, setFileFilter, setFileHidingEnabled, setFileSelectionMode, setFileSystemView, setFileView, setMultiSelectionEnabled, setSelectedFile, setSelectedFiles, updateUI`

**Methods inherited from class javax.swing.JComponent**

`addAncestorListener, addNotify, addVetoableChangeListener, computeVisibleRect, contains, createToolTip, disable, enable, firePropertyChange, firePropertyChange, firePropertyChange, getActionForKeyStroke, getActionMap, getAlignmentX, getAlignmentY, getAncestorListeners, getAutoscrolls, getBaseline, getBaselineResizeBehavior, getBorder, getBounds, getClientProperty, getComponentPopupMenu, getConditionForKeyStroke, getDebugGraphicsOptions, getDefaultLocale, getFontMetrics, getGraphics, getHeight, getInheritsPopupMenu, getInputMap, getInputMap, getInputVerifier, getInsets, getInsets, getListeners, getLocation, getMaximumSize, getMinimumSize, getNextFocusableComponent, getPopupLocation, getPreferredSize, getRegisteredKeyStrokes, getRootPane, getSize, getToolTipLocation, getToolTipText, getToolTipText, getTopLevelAncestor, getTransferHandler, getVerifyInputWhenFocusTarget, getVetoableChangeListeners, getVisibleRect, getWidth, getX, getY, grabFocus, isDoubleBuffered, isLightweightComponent, isManagingFocus, isOpaque, isOptimizedDrawingEnabled, isPaintingForPrint, isPaintingTile, isRequestFocusEnabled, isValidateRoot, paint, paintImmediately, paintImmediately, print, printAll, putClientProperty, registerKeyboardAction, registerKeyboardAction, removeAncestorListener, removeNotify, removeVetoableChangeListener, repaint, repaint, requestDefaultFocus, requestFocus, requestFocus, requestFocusInWindow, resetKeyboardActions, reshape, revalidate, scrollRectToVisible, setActionMap, setAlignmentX, setAlignmentY, setAutoscrolls, setBackground, setBorder, setComponentPopupMenu, setDebugGraphicsOptions, setDefaultLocale, setDoubleBuffered, setEnabled, setFocusTraversalKeys, setFont, setForeground, setInheritsPopupMenu, setInputMap, setInputVerifier, setMaximumSize, setMinimumSize, setNextFocusableComponent, setOpaque, setPreferredSize, setRequestFocusEnabled, setToolTipText, setTransferHandler, setVerifyInputWhenFocusTarget, setVisible, unregisterKeyboardAction, update`

**Methods inherited from class java.awt.Container**

`add, add, add, add, add, addContainerListener, addPropertyChangeListener, addPropertyChangeListener, applyComponentOrientation, areFocusTraversalKeysSet, countComponents, deliverEvent, doLayout, findComponentAt, findComponentAt, getComponent, getComponentAt, getComponentAt, getComponentCount, getComponents, getComponentZOrder, getContainerListeners, getFocusTraversalKeys, getFocusTraversalPolicy, getLayout, getMousePosition, insets, invalidate, isAncestorOf, isFocusCycleRoot, isFocusCycleRoot, isFocusTraversalPolicyProvider, isFocusTraversalPolicySet, layout, list, list, locate, minimumSize, paintComponents, preferredSize, printComponents, remove, remove, removeAll, removeContainerListener, setComponentZOrder, setFocusCycleRoot, setFocusTraversalPolicy, setFocusTraversalPolicyProvider, setLayout, transferFocusBackward, transferFocusDownCycle, validate`

**Methods inherited from class java.awt.Component**

`action, add, addComponentListener, addFocusListener, addHierarchyBoundsListener, addHierarchyListener, addInputMethodListener, addKeyListener, addMouseListener, addMouseMotionListener, addMouseWheelListener, bounds, checkImage, checkImage, contains, createImage, createImage, createVolatileImage, createVolatileImage, dispatchEvent, enable, enableInputMethods, firePropertyChange, firePropertyChange, firePropertyChange, firePropertyChange, firePropertyChange, getBackground, getBounds, getColorModel, getComponentListeners, getComponentOrientation, getCursor, getDropTarget, getFocusCycleRootAncestor, getFocusListeners, getFocusTraversalKeysEnabled, getFont, getForeground, getGraphicsConfiguration, getHierarchyBoundsListeners, getHierarchyListeners, getIgnoreRepaint, getInputContext, getInputMethodListeners, getInputMethodRequests, getKeyListeners, getLocale, getLocation, getLocationOnScreen, getMouseListeners, getMouseMotionListeners, getMousePosition, getMouseWheelListeners, getName, getParent, getPeer, getPropertyChangeListeners, getPropertyChangeListeners, getSize, getToolkit, getTreeLock, gotFocus, handleEvent, hasFocus, hide, imageUpdate, inside, isBackgroundSet, isCursorSet, isDisplayable, isEnabled, isFocusable, isFocusOwner, isFocusTraversable, isFontSet, isForegroundSet, isLightweight, isMaximumSizeSet, isMinimumSizeSet, isPreferredSizeSet, isShowing, isValid, isVisible, keyDown, keyUp, list, list, list, location, lostFocus, mouseDown, mouseDrag, mouseEnter, mouseExit, mouseMove, mouseUp, move, nextFocus, paintAll, postEvent, prepareImage, prepareImage, remove, removeComponentListener, removeFocusListener, removeHierarchyBoundsListener, removeHierarchyListener, removeInputMethodListener, removeKeyListener, removeMouseListener, removeMouseMotionListener, removeMouseWheelListener, removePropertyChangeListener, removePropertyChangeListener, repaint, repaint, repaint, resize, resize, setBounds, setBounds, setComponentOrientation, setCursor, setDropTarget, setFocusable, setFocusTraversalKeysEnabled, setIgnoreRepaint, setLocale, setLocation, setLocation, setName, setSize, setSize, show, show, size, toString, transferFocus, transferFocusUpCycle`

**Methods inherited from class java.lang.Object**

`equals, getClass, hashCode, notify, notifyAll, wait, wait, wait`

**Field Detail**

### UNHANDLED\_DIALOG

```
public static final int UNHANDLED_DIALOG
```

:   unhandled type of dialog

    **See Also:**: Constant Field Values

---


### LOADER\_DIALOG

```
public static final int LOADER_DIALOG
```

:   the loader dialog

    **See Also:**: Constant Field Values

---


### SAVER\_DIALOG

```
public static final int SAVER_DIALOG
```

:   the saver dialog

    **See Also:**: Constant Field Values


**Constructor Detail**

### ConverterFileChooser

```
public ConverterFileChooser()
```

:   onstructs a FileChooser pointing to the user's default directory.

---


### ConverterFileChooser

```
public ConverterFileChooser(java.io.File currentDirectory)
```

:   Constructs a FileChooser using the given File as the path.

    **Parameters:**: `currentDirectory` - the path to start in

---


### ConverterFileChooser

```
public ConverterFileChooser(java.lang.String currentDirectory)
```

:   Constructs a FileChooser using the given path.

    **Parameters:**: `currentDirectory` - the path to start in


**Method Detail**

### setCapabilitiesFilter

```
public void setCapabilitiesFilter(Capabilities value)
```

:   sets the capabilities that the savers must have. use null if all should be
    listed.

    :   **Parameters:**: `value` - the minimum Capabilities the savers must have

---


### getCapabilitiesFilter

```
public Capabilities getCapabilitiesFilter()
```

:   returns the capabilities filter for the savers, can be null if all are
    listed.

    :   **Returns:**: the minimum Capabilities the savers must have

---


### setOverwriteWarning

```
public void setOverwriteWarning(boolean value)
```

:   Whether a warning is popped up if the file that is to be saved already
    exists (only save dialog).

    :   **Parameters:**: `value` - if true a warning will be popup

---


### getOverwriteWarning

```
public boolean getOverwriteWarning()
```

:   Returns whether a popup appears with a warning that the file already
    exists (only save dialog).

    :   **Returns:**: true if a warning pops up

---


### setFileMustExist

```
public void setFileMustExist(boolean value)
```

:   Whether the selected file must exst (only open dialog).

    :   **Parameters:**: `value` - if true the file must exist

---


### getFileMustExist

```
public boolean getFileMustExist()
```

:   Returns whether the selected file must exist (only open dialog).

    :   **Returns:**: true if the file must exist

---


### setCoreConvertersOnly

```
public void setCoreConvertersOnly(boolean value)
```

:   Whether to display only the hardocded core converters. Necessary for
    RMI/Remote Experiments (dynamic class discovery doesn't work there!).

    :   **Parameters:**: `value` - if true only the core converters will be displayed **See Also:**: `m_CoreConvertersOnly`

---


### getCoreConvertersOnly

```
public boolean getCoreConvertersOnly()
```

:   Returns whether only the hardcoded core converters are displayed.
    Necessary for RMI/REmote Experiments (dynamic class discovery doesn't
    work there!).

    :   **Returns:**: true if the file must exist **See Also:**: `m_CoreConvertersOnly`

---


### showDialog

```
public int showDialog(java.awt.Component parent,
                      java.lang.String approveButtonText)
```

:   Pops a custom file chooser dialog with a custom approve button. Throws
    an exception, if the dialog type is UNHANDLED\_DIALOG.

    :   **Overrides:**: `showDialog` in class `javax.swing.JFileChooser`
    :   **Parameters:**: `parent` - the parent of this dialog: `approveButtonText` - the text for the OK button **Returns:**: the user's action

---


### showOpenDialog

```
public int showOpenDialog(java.awt.Component parent)
```

:   Pops up an "Open File" file chooser dialog.

    :   **Overrides:**: `showOpenDialog` in class `javax.swing.JFileChooser`
    :   **Parameters:**: `parent` - the parent of this file chooser **Returns:**: the result of the user's action

---


### showSaveDialog

```
public int showSaveDialog(java.awt.Component parent)
```

:   Pops up an "Save File" file chooser dialog.

    :   **Overrides:**: `showSaveDialog` in class `javax.swing.JFileChooser`
    :   **Parameters:**: `parent` - the parent of this file chooser **Returns:**: the result of the user's action

---


### getLoader

```
public AbstractFileLoader getLoader()
```

:   returns the loader that was chosen by the user, can be null in case the
    user aborted the dialog or the save dialog was shown

    :   **Returns:**: the chosen loader, if any

---


### getSaver

```
public AbstractFileSaver getSaver()
```

:   returns the saver that was chosen by the user, can be null in case the
    user aborted the dialog or the open dialog was shown

    :   **Returns:**: the chosen saver, if any

---


### main

```
public static void main(java.lang.String[] args)
                 throws java.lang.Exception
```

:   For testing the file chooser

    :   **Parameters:**: `args` - the commandline options - ignored **Throws:**: `java.lang.Exception` - if something goes wrong with loading/saving


---


|  |  |  |
| --- | --- | --- |
| **Overview** \| **Package** \| **Class** \| **Tree** \| **Deprecated** \| **Index** \| **Help** \| **Weka's home** | |  |
| **PREV CLASS**   **NEXT CLASS** | **FRAMES**    **NO FRAMES**     **All Classes** |  |
| SUMMARY: NESTED \| FIELD \| CONSTR \| METHOD | DETAIL: FIELD \| CONSTR \| METHOD |  |


---
